# Peritoneal dialysis after shared decision-making: the disparity between reality and patient expectations

**DOI:** 10.1186/s12912-022-01043-5

**Published:** 2022-09-30

**Authors:** Ya-Fang Ho, Pei-Ti Hsu, Kai-Ling Yang

**Affiliations:** 1 School of Nursing, China Medical University, No. 100, Sec. 1, Jingmao Rd., Beitun Dist, 406040 Taichung City, Taiwan, ROC; 2 Department of Nursing, Ching Kuo Institute of Management and Health, Keelung, Taiwan, ROC; 3 Nursing Supervisor, Nephrology Medicine, China Medical University Hospital, Taichung, Taiwan, ROC

**Keywords:** Peritoneal dialysis, Renal replacement therapy, Shared decision-making, Quality of life

## Abstract

**Background:**

The current health policy in Taiwan favors peritoneal dialysis (PD) at home. Policy objectives may make healthcare providers give more consideration to the introduction of PD treatment. This study aimed to explore the process of information acquisition and consideration during shared decision-making (SDM) for patients undergoing PD and compare their quality of life expectations before and after PD at home.

**Methods:**

In this qualitative study, 15 patients undergoing PD for < 12 months were purposively recruited from one large PD unit in Taichung, Taiwan. Data were collected between August 2020 and December 2020 using a semi-structured interview. All transcripts were evaluated using thematic analysis.

**Results:**

Three themes and seven subthemes were identified following data analysis: 1. sources for information on dialysis treatment, including (a) effect of others’ experiences and (b) incomplete information from healthcare providers (HCPs); 2. considerations for choosing PD, including (a) trusting physicians, and (b) maintaining pre-dialysis life; and 3. disparity between pre-and post-PD reality and expectation, including (a) limitation by time and place, (b) discrepancies in expected freedom and convenience, and (c) regret versus need to continue.

**Conclusion:**

HCPs played an important role in SDM, providing key information that influenced the process. Patients undergoing initial PD at home exhibited a disparity between expectation and reality, which was exacerbated by incomplete information.

## Background

For patients with end-stage renal disease (ESRD), the choice of treatment is a major decision, which will significantly affect patients’ quality of life [1]. Renal replacement therapy (RRT) is the main life-sustaining treatment for patients with ESRD, with in-center hemodialysis (ICHD) and home-based peritoneal dialysis (PD) being the most common forms in Taiwan.

Relevant studies have pointed out that patients undergoing PD have a better quality of life (QoL) and higher survival rate than patients undergoing ICHD [2, 3]. However, some studies have also pointed out that there were no statistically significant differences in QoL and survival rate between patients undergoing PD and those undergoing ICHD [4, 5]. The difference between QoL and survival rate in ICHD and PD is unclear. The advantage of PD over ICHD is that PD can be performed at home by patients, without the need to travel to a hemodialysis center, with increased flexibility and freedom. Accumulating evidence demonstrated that PD has been a cost-saving treatment compared to ICHD in most developed countries and some developing countries [4, 6]. The current policy in Taiwan tends to be “PD-Favored”, which means the government’s dialysis policy encourages the use of PD as the treatment option while removing any existing disincentives [4]. This policy incentivizes healthcare professionals (HCPs), such as nephrologists and nephrology nurses, to give more consideration to the introduction of PD treatment at home. The related studies have highlighted that compared with patients undergoing HD, patients undergoing PD get more adequate information before dialysis [7–9]. One factor may be that patients undergoing PD are usually younger or more educated; however, it is undeniable that this may also be related to the patients receiving more information about PD [10, 11].

In recent years, an important part of care is to engage patients in their medical decision-making [12]. Shared decision-making (SDM) is regarded as a strategy to realize patient-centered care that is considered a key factor in achieving a better quality of life after starting dialysis [1]. In the information exchange stage of SDM, HCPs must provide professional knowledge for all treatment options required [13]. The information provided by HCPs at this stage will guide the patient’s decision on dialysis treatment. Incomplete or biased information on the options will affect the patient’s choice of dialysis method [14, 15]. A study has found that HCPs may offer PD as an option more often to patients with higher health literacy or better self-care abilities [7]. Although PD emphasizes flexibility and freedom as its advantages, renal replacement therapy must be tailored to the specific needs and preferences of the patient [6, 16]; thus, PD is not always the most appropriate treatment modality for the patient. Inadequate patient education might result from the candidacy bias of physicians and nurses [17]. Driven by the current “PD-Favored” policy, we observed that Taiwanese nephrologists might incline to recommend PD to patients “they think” is suitable. However, patients with ESRD focus on the type of dialysis treatment that can maintain their pre-dialysis lifestyle, requiring sufficient information to understand treatment [16]. Incomplete information may cause a patient’s disparity between expectations and reality after initiation of PD. Because the effect of PD on patients is not only the treatment of the disease but also the problem of coordinating dialysis treatment with other aspects of daily life, such as work, based on our clinical experience, if the patient was unable to cope with the relevant problems, the PD treatment may become a completely negative experience. Although many studies have explored the decision-making considerations of patients with ESRD in the shared decision-making (SDM) process [18–20], to the best of our knowledge, few studies have explored whether PD patients’ dialysis life after an SDM meets the patient’s expectations of maintaining their pre-dialysis lifestyle. Therefore, this study aimed to explore the process of information acquisition and consideration during SDM for patients undergoing PD and compare their QoL expectations before and after PD at home.

## Methods

### Design

This was a qualitative descriptive study. We conducted semi-structured interviews with adults who were receiving PD for ESRD from August 2020 to December 2020. To maintain confidentiality, all participants were assigned numbers, and all identifying data have been excluded. We followed the consolidated criteria for reporting qualitative research guidelines (COREQ).

### Participants and recruitment

Fifteen participants were recruited from one large PD unit in Taichung, Taiwan. We used purposive sampling to enroll patients with ESRD who are undergoing PD for < 12 months, are aged > 20 years, could communicate in Mandarin or Taiwanese language, and could provide informed consent. All participants underwent the hospital’s one-time SDM process before starting dialysis. The SDM process included a meeting of the patient with their physician to discuss disease progression and a 1-to-1 session with nephrology nurses. Written and audiovisual decision aids were used at sessions, and written information was provided for patients to take home. According to the patient list of the PD center, the interviewer contacted potential participants, explained the study, and invited them to participate. If people agreed to participate, written informed consent was obtained before the interview began.

### Data collection

A preliminary interview topic guide was developed from discussion with the research team. The final interview guide is provided in Table 1. The duration of the interview with the patients was 30–40 min. The interviews were undertaken in the hospital, usually in a private room, and were scheduled according to patient preference. The primary researcher (author 1), who had a Ph.D. and received qualitative interview training, conducted all interviews to maintain consistency. Translation was not required during the interview process as the primary researcher is proficient in the Taiwanese and Mandarin language. All interviews were audiorecorded and transcribed verbatim by a professional transcription company. Fieldwork notes were taken by the primary researcher (interviewer) to capture participants’ feelings, emotions, and nonverbal expressions. The data collection and analysis were iterative; recruitment was terminated when no new descriptive themes emerged in the analysis.

**Table 1:** Interview questions

Item	Interview questions
1	Do you think your life after dialysis was the same as what you expected? Have you ever faced any difficulties?
2.	Did the doctors and/or nurses give you any information or advice when you made a choice?
3	Can you talk about how you felt when the doctors and/or nurses give you information or advice?
4	How well did you understand all the given information?
5	Did you obtain information from sources (e.g., Internet and books) other than your doctor or nurse?
6	What were your considerations when you made dialysis mode choices?
7	Do you think you made the right decision? Why?

### Data analysis

We inductively analyzed all 15 patient transcripts using thematic analysis [21]. Two researchers (authors 1 and 2) independently reviewed the transcripts word-by-word to capture crucial concepts and were given an initial code (Table 2). After the initial coding, all the codes were compared and summarized into subthemes according to their conceptual similarity. Three researchers met and discussed after coding the first five transcripts to finalize the coding scheme, and the agreed-upon codes and subthemes were systematically applied to all subsequent transcripts. Afterward, the primary researcher (author 1) coded the subsequent data and met with two other researchers every month to discuss it. Finally, the subthemes were categorized under main themes through discussion by all researchers. The fieldwork notes were consulted during analysis to aid final interpretations. NVivo12 was used to manage the data. The use of NVivo12, transcript sharing, and data discussions supported a transparent approach to working with data and ensured that the final themes were robust.

**Table 2:** Examples of abstraction from transcript to codes

Identification of meaningful phrases in transcript	Condensed phrases	Description	Codes
With exchange of the dialysate 4–5 times a day, I feel like my life…just waiting for the dialysate change (5–37)…., My time is tied by dialysis (5–80). Before dialysis, I thought PD was freedom, but after starting dialysis, I found that time was bound by the exchange of dialysate (5–134). *(NO.5)* I spend a lot of time in the dialysate change a day, which takes up a lot of my day, and I can’t take a break (7–35) …I felt that my time was occupied (7–37). It feels like nothing has been done; then, I must change the dialysate (7–38). (*NO.7*)	5–37 Exchange of dialysate is my whole daily life 5–80 My time is tied by PD 5–134 The time is bound by the exchange of dialysate 7–35 Dialysate exchange takes up time 7–37 My time is occupied 7–38 Daily life is left with only dialysis	Life is bound by dialysis	Trapped by PD
My nurse said that PD must be done 4 times a day. There is not even one less, so once the time is up, I have to change the dialysate (15–278). (*NO.15*) I feel that time is tied, and I feel like I am waiting to do this thing (exchange of dialysate) every day (12–194). I don’t have a long time to do something else…… I have to cooperate with the time of changing the dialysate (12–197). (*NO.12*)	15–278 I had to change the dialysate on time. 12–194 Waiting for exchange of dialysate every day. 12–197 Cooperate with the time of dialysate exchange.	I’m waiting to change the dialysate every day	

### The rigor of the study

The credibility was enhanced by comprehensive field notes, peer debriefing, and transcription rigor. We randomly selected one interview record for a peer debriefing, and the two researchers then compared and discussed their interpretations of the data until a consensus was reached on the interpretation that was most consistent with the original meaning of the data. The field notes captured the participants’ feelings, emotions, and nonverbal expressions that assisted in the credibility of the analysis data. All team members have received qualitative research training, which increases rigor in the transcription of data. Dependability was achieved using the standard procedure of the thematic analysis approach in data analysis, and the three researchers discussed every month until a consensus was reached on the interpretation most consistent with the original meaning of the data. Transferability was assured by generating detailed descriptions of the study methodology, sample selection criteria, data collection process, saturation of the data, and context. Furthermore, the confirmability of the findings was secured by team members checking the associated notes and text of the original data to ensure that the interpretations of the findings were not influenced by personal opinions and biases.

### Ethical considerations

The study was approved by the research ethics committee of an academic medical center in Taichung city (CMUH109-REC3-085). We obtained the patient’s written informed consent prior to the interview.

## Results

Among the 20 participants who were approached, five declined. Two of them were unable to cooperate due to outpatient time, the other two refused to be interviewed, and one was unwell in the interview. The demographic characteristics of the 15 participants are shown in Table 3. The patients interviewed were 21–75 years old. Most participants were male (80%), six (40%) were unemployed, six (40%) were employed, and five (33%) had a university level of education. The time since dialysis started ranged from 2 months to 12 months. Three main themes were identified following data analysis and are discussed in the succeeding sections. A list of the themes, subthemes, and codes is provided in Table 4.

**Table 3:** Participant characteristics (n = 15)

Demographics	n (%)
Age	
Younger than 40	4(27%)
40–49	2(13%)
50–59	3(20%)
60–69	4(27%)
70 or older	2(13%)
Gender	
Male	12(80%)
Female	3(20%)
Employment status	
Unemployed	6(40%)
Employed	6(40%)
Retirement	3(20%)
Education level	
Elementary school	2(13%)
Junior high school	3(20%)
High school	4(27%)
College school	1(7%)
University	5(33%)
Time on dialysis	
0–3 months	4(27%)
3–6 months	4(27%)
6–12 months	7(46%)

**Table 4:** List of the themes, subthemes, and codes

Theme	Subtheme	Code	Description
**Sources for information on dialysis treatment**	Effect of others’ experiences	Sick friend’s experience	Feeling of being understood We have the same experience
		Dialysis experience	Real experience from family or friends Positive or negative information about dialysis
	Incomplete information from HCPs	Only talk about the benefits of PD	Only talks about the advantages of PD I don’t know the risks of PD
		Focus on the PD	Only focus on PD Don’t explain the other dialysis mode
		Suitable for my life	Impact of dialysis on life A good choice is suitable for the current life
**Considerations for choosing PD**	Trusting physicians	Trust physician	I trust my physician The physician is a professional
		Physician recommends	PD is best for me I agree with the physician’s advice
	Maintaining pre-dialysis life	Keep normal life	Not life-changing is the key Keep daily life is my concern
		Cooperate with work	Dialysis doesn’t affect work Convenience is my consideration
**Disparity between pre-and post-PD reality and expectation**	Limited by time and place	Trapped by PD	Life is bound by dialysis I’m waiting to change the dialysate every day
		Limitations in the workplace	PD requires a separate space No time to change dialysate at work
		Restrictions on going out	No separate space The environment is not clean I’m worried about getting infected
	Discrepancies in expected freedom and convenience	Cooperate with PD	I have to adjust my life Life must cooperate with PD
		No freedom	There is no more freedom Not as convenient as imagined
		Not my expectation	Different from what I thought Conflicted with current experience
	Regret versus need to continue	Regret	I regret it This is not the life I expected
		Keep doing	I have to keep doing Forced to maintain this way

## Sources for information on dialysis treatment

Overall, the participants stated that, in the process of SDM discussion, they all received valuable information (e.g., educational courses, videos, and written materials) to help their decision-making. Most participants felt that the physicians and nurses were professional and caring. However, in the choice of dialysis method, in addition to actual experience, physicians and nurses had a significant influence on their choice, with a preference for choosing PD.

### Effect of others’ experiences

It is common to seek knowledge from other sources, such as the Internet, friends, and family members with dialysis experience. In addition to the information provided by HCPs, younger patients use the Internet to find relevant information. Moreover, there is misinformation about dialysis from family members or relatives and friends. Actual experience can affect the patient’s choice of dialysis method, especially experience from family or friends, and vicarious learning through the experiences of others. Positive actual experiences tend to make patients choose the same method, whereas negative actual experiences also make patients reject this dialysis method.

*If I really have to dialysis, I will choose peritoneal dialysis, because my wife is doing peritoneal dialysis……My friend died a month after doing hemodialysis……, anyway I don’t dare to do hemodialysis. (NO.2)*

Some participants believe that communication between patients would allow them to meet their needs better, and their PD treatment experience-sharing provides participants the feeling of sympathy and understanding.

*The sick friend shared his PD treatment experience with me, I felt better, I would think PD treatment was really nothing… after all, and he will know what I need. (NO.14)*

### Incomplete information from HCPs

Most participants reported that physicians preferred PD in their recommendations regarding dialysis methods, especially for younger patients. Regarding the advantages and disadvantages of each dialysis method, HCPs should provide clear information. However, during the SDM process, they felt that HCPs showed a preference for PD and emphasized the advantages of PD in the explanation, avoiding the risks and disadvantages. HCPs emphasized that PD is flexible and has the advantages of freedom and convenience that allow patients to maintain work.

*Although the nurse will introduce each method, I felt….she was more inclined to the peritoneal dialysis… she said the many benefits of peritoneal dialysis, such as freedom and convenience…….but, you(nurse) must clearly explain what will happen, didn’t just incline to introduce more peritoneal dialysis. (NO.7)*

Most participants believe that HCPs provided impetus to choose PD as they provided participants with an explanation of the dialysis method they felt was most appropriate and tended to encourage participants to choose it. When a patient revealed his inclination to do PD, HCPs were inclined to provide more information about PD, while ignoring other treatment options, and believed that this was based on the patient’s values and preferences.

*The physician advised me to do peritoneal dialysis, so the nurses told me about peritoneal dialysis a lot, and hemodialysis said very little…. The nurse didn’t talk about kidney transplantation in-depth, maybe because I was not suitable for it. (NO.8)*

## Considerations for choosing PD

### Trusting physicians

The participants thought that although the physician provided an explanation about dialysis, they did not always understand what the physician said. When recalling experiences of how they had been involved in decisions about treatment modality, most participants positioned themselves as passive and initiated PD dialysis through the treatment recommended by the physician. Some participants felt that they did not really decide but rather agreed with the physician’s advice. Physicians are regarded as experts with professional knowledge by the participants, and they trust the physician to make the “correct” decision on their behalf. The participants talked about trust in the expertise of physicians and expressed their belief that any advice given by the physician will bring the best results. They were willing to follow the physician’s advice on the choice of dialysis method.

*I trust the doctor. Actually, I don’t understand some of what the doctor said, but I just trust him. (NO.3)*

*I trust the doctor. I didn’t know which dialysis method was better….I was not a professional. The doctor suggested doing peritoneal dialysis, just listen to the doctor…. I think what he said should be good. (NO.15)*

### Maintaining pre-dialysis life

When faced with the choice of dialysis treatment, most participants said that the decision was influenced by the expectation of “maintaining the original lifestyle,” and the impact of dialysis treatment on their lives was their focus.

*The point is the impact of this method I chose on my life because after all, dialysis is a problem for my life. Not changing my lifestyle is the focus of my consideration…. (NO.6)*

The participants’ decision-making focused on which dialysis treatment has less effect on their current life for living a normal life was a key factor in most participants’ decisions. They hoped that dialysis would not change their lifestyle and would not interfere with their work. PD was the closest approach to their expectation of a “normal life.”

*I wanted to know which kind of treatment would not affect my daily life…. the life of a normal person is the key to my decision. Convenience is my biggest consideration… if I can do it myself, it would be better, and it will not affect my work. (NO.13)*

## Disparity between pre-and post-PD reality and expectation

### Limited by time and place

After dialysis, most participants found that replacing the dialysate was time-consuming. Even if they were going out, they took the time to replace the dialysate, leaving less time for other activities.

*I spend a lot of time in the dialysate change a day, which takes up a lot of my day, and I can’t take a break….I felt that my time was occupied…. it feels like nothing has been done, and then I must change the dialysate. (NO.7)*

Some participants considered the outside environment as unsafe. Even when going with the dialysate, finding a clean and independent space to replace the dialysate was difficult. They avoided going out because they were worried about infection during the dialysate replacement process.

*We are worried about infection, so we dare not change dialysate outside, and we must go home when the time is up. ….The nurse said that peritoneal dialysis can be changed dialysate everywhere, but in fact, we don’t dare, we will worry about infection. (NO.14)*

If the workplace cannot provide a separate space for replacing the dialysate, returning to work may not be possible. For example, a participant who was originally engaged in civil engineering said that he needed to work on the construction site, and the construction site did not have a suitable space to perform the dialysis. This dilemma caused him to lose his job. Even though he adopted automated peritoneal dialysis, he still could not avoid the problem that he needs to replace the dialysate at noon.

*I can’t go to work because I have to be outside on the construction site, even I do automated peritoneal dialysis at night, I also don’t have a place to change dialysate at noon. (NO.11)*

### Discrepancies in expected freedom and convenience

Many participants described the need to coordinate their lives to match the efforts and challenges of replacing the dialysate. The participants said that before dialysis, they felt that PD should not be troublesome. After dialysis, they found that it was not as free and convenient as expected because the dialysate must be replaced at a fixed time; their lives and work must be changed to accommodate the dialysis time. One participant said,

*I must adjust my life with the time of changing dialysate…. if oversleeping will disrupt the time of changing dialysate. …. peritoneal dialysis is different from what I originally thought, there is no more freedom. (NO.5)*

Another participant who used automated peritoneal dialysis said that replacing the dialysate at noon prevented her from having lunch with colleagues or even taking a break at noon.

*I can’t have lunch with my colleagues at noon because I must change the dialysate… I think (automated peritoneal dialysis) is different as I originally thought. (NO.4)*

Although the participants emphasized the importance of freedom, flexibility, work maintenance, and the maintenance of their original life, these factors often conflicted with their current dialysis experience. The various inconveniences in life made the participants aware of the gap between expectations and reality.

*After starting dialysis, I felt that peritoneal dialysis was not as convenient as expected….Sometimes you go out to play, it may take several hours. Then you must catch the fixed time for dialysis…It’s different from before imagination. (NO.10)*

### Regret versus need to continue

Some participants expressed regret after starting PD because it was different from the “normal life” they expected. They should continue the treatments even if they felt the choice was wrong. One participant stated that they had an intraperitoneal catheter implantation operation and had to compromise and continue to receive PD. The participants described their disappointments with PD and how they accepted and were forced to adapt to the PD treatment, regardless of their initial expectations of the treatment.

I want to say that I chose…this choice was wrong…I regret it. (NO.5)

*In order to survive, no matter whether the choice is right or not, you have to keep doing….I feel regret after peritoneal dialysis, but I can only be forced to maintain this way. (NO.4)*

## Discussion

### Source and incompleteness of information

This study found that patients obtain information about dialysis from family members or relatives and friends. Some participants had the actual dialysis experience of family or friends for alternative learning. Furthermore, the patient believed that sick friends who had encountered the same experience were more empathetic. This was similar to the findings of Morton et al. [22], Griva et al. [23], and Harwood and Clark [19]. Social influences, such as hearsay, family involvement, and experiences of others, can hinder or promote the choice of dialysis method. The patient’s choice of dialysis method was greatly influenced by the positive or negative experiences of other patients. Highly personalized stories can be viewed as persuasive and manipulative, and narratives can unintentionally present as biased or unbalanced information, which may influence decision-making [24]. Although the current literature does not support the narratives to be a required element of decision aids, the narrative may play a key role in “feeling supported,” whereas experience narratives designed to comfort could contribute to the goals of decision aids [25]. Syrowatka et al. have highlighted that support from others who faced the same decision should be integrated into decision aids for patients to feel not alone in their experience or decision-making [26]. Therefore, it is recommended that when using patient stories, HCPs should avoid including subjective testimonials about treatment and focus on how to face dialysis and share the mental journey of choosing different treatments, which can help patients obtain empathetic psychological support.

The study found that HCPs had a great influence on the choice of dialysis method for patients. HCPs focused on the advantages of PD, emphasizing that it was a flexible and autonomous treatment method, which was similar to the results of Harwood and Clark [19]. Their study has found that if HCPs believed that the old people were unsuitable for home dialysis, they would try to influence the patient’s choice. This study found that HCPs constructed information about treatment options in a way that guided patients to adopt specific dialysis methods. Taiwan’s related policies advocate dialysis treatment with PD as the priority. This may cause HCPs to downplay the shortcomings of this treatment method or to overstate the advantages of PD, which is likely to produce patient bias in the absorption of information. They will tend to evaluate the pros and cons of PD treatment, rather than weighing the pros and cons between different treatment modalities. Perhaps from the viewpoint of HCPs, incomplete information on the options does not mean that they did not discuss the choice of dialysis method with the patient. Himmelfarb et al. pointed out that the views of HCPs do not always align with those of patients and their families, and HCPs are often not well prepared for SDM [17]. Although SDM was concerned about the autonomy of patient choice, HCPs lacked an SDM understanding of patient participation, which may cause HCPs to perceive that they were facilitating it while still operating in a traditional work frame. The unequal professional knowledge in the HCPs–patient relationship may result in the choice of a specific dialysis method preference and lose the meaning of SDM to make the most suitable treatment choice [27].

## Considerations for choosing PD

As in previous studies results, maintaining their pre-dialysis lifestyle was the most important factor for patients with ESRD to choose the dialysis mode in our study. However, we have found that the relationship between physicians and patients had a great influence on the selection of PD. The meta-analysis by Shi et al. has highlighted that the relationship between the patient and the physician was quite complicated, including the asymmetry of trust and power, which will affect the patient’s treatment decision [28]. Trust can be a facilitating or hindering factor in SDM because it may cause the patient to become passive in SDM [29, 30]. When the physician’s implicit expectation and patients’ treatment expectations are not the same, the patient may find it challenging to express different opinions or objections and worry about the damage to their relationship with their physician [10, 29]. Equal partnership between patients and HCPs is one of the main concepts of SDM [13]. However, the relationship between patients and physicians in the clinical setting creates power asymmetry, and physicians have duality in the decision-making process. On the one hand, physicians emphasized the patient’s individual choice; on the other hand, they influenced decision-making by advocating certain treatments; the way they present information can lead patients to choose specific treatments [27]. Therefore, physicians should pay attention to understanding how they influence the choice of patients’ dialysis method in SDM, especially when providing opinions and guarantees on their choice, and HCPs should make good use of a mutual trusting relationship to help form SDM. Establishing a trusting relationship encourages patients to share their concerns and preferences and ask questions courageously, and when healthcare providers assist patients in solving problems, this trust will increase. This creates a positive cycle, thereby indirectly improving patients’ decision-making ability.

## The disparity between reality and expectation

This study found that patients who have just started PD have experienced a disparity between pre-and post-PD expectations and reality and are facing coordination and adaptation problems after starting PD. The expectation when obtaining information before dialysis was in sharp contrast with reality after starting dialysis. Incomplete or biased information on the treatment options by HCPs exacerbates this disparity between expectation and reality. Patients found that PD violates the expectation that life will not be affected, it was not the freedom and convenience as expected, and it was still limited by time and place. Patients felt that they were tied to life by PD and regretted their original choice. This was different from other studies that indicated that patients were satisfied with the choice of PD after dialysis [7, 18]. Perhaps, because of the initial PD, patients must coordinate and adapt in life and dialysis practice and often encounter difficulties and frustrations. Because of the operational requirements of PD, it will inevitably cause some restrictions on the life of the patient. These isolation requirements and operations are potentially important threats to the maintenance of patients’ social life, and these patients often do not realize the impact of dialysis on their lives before dialysis, and HCPs had not mentioned it. Winterbottom et al. have found that when facing the choice of dialysis method, patients can only imagine the impact of their choice of dialysis mode on their future lives [31]. When the patient regrets the inability to cooperate and deal with related problems after entering PD treatment, the coordination between daily life and dialysis treatment will continue to be frustrating. Therefore, HCPs should provide complete information on treatment options during the information exchange stage. In addition, HCPs also must think about how to provide assistance to patients undergoing initial PD with different life backgrounds, helping patients safely and flexibly coordinate between the operation of PD and life.

## Limitations

Our study results should be used cautiously, the representativeness of patients may be limited because we only included patients currently admitted to PD centers. Second, the recruitment of participants was voluntary, so those who refuse to participate may also have different opinions. Due to the effective control of the epidemic by the Taiwanese government, the COVID-19 pandemic had not affected our recruitment and data collection. The first author has previously worked as a dialysis nurse in a dialysis center for 20 years, and her extensive field knowledge was valuable in conducting the study. However, field knowledge comes with potentially preconceived notions, and co-authors from other backgrounds could provide new interpretations of research findings.

## Conclusion

This study explored the experience of information acquisition and consideration that PD patients in the SDM process, and compare their quality of life expectations before and after PD at home. The participants said that HCPs have a significant impact on their choice. This was demonstrated in the patient’s description of their PD decision factors. Although SDM was concerned about the autonomy of patient choice, HCPs lacked an SDM understanding of patient participation. Appropriate education and training of HCPs could help to solve this problem. Furthermore, patients with initial PD have experienced a disparity between pre-and post-PD expectations and reality. Incomplete information on the treatment options by HCPs exacerbates this disparity between expectation and reality. Therefore, HCPs providing accurate and unbiased information to support patient decision-making is especially relevant.

## Data Availability

The datasets used and analyzed during the present study are available from the corresponding author on reasonable request.

## List of abbreviations

ESRD: End-stage renal disease.
HCPs: Help healthcare providers.
ICHD: In-center hemodialysis.
PD: Peritoneal dialysis.
QoL: Quality of life.
RRT: Renal replacement therapy.
SDM: Shared decision-making.
